# A Concise Account of Information as Meaning Ascribed to Symbols and Its Association with Conscious Mind

**DOI:** 10.3390/e25010177

**Published:** 2023-01-16

**Authors:** Yunus A. Çengel

**Affiliations:** Department of Mechanical Engineering, University of Nevada, Reno, NV 89557, USA; yunus.cengel@yahoo.com

**Keywords:** information, knowledge, meaning, mind, consciousness, symbols of information, transmission of information

## Abstract

The term *information* is used in different meanings in different fields of study and daily life, causing misunderstanding and confusion. There is a need to clarify what information is and how it relates to *knowledge*. It is argued that information is *meaning* represented by *physical symbols* such as sights, sounds, and words. Knowledge is meaning that resides in a conscious mind. The basic building blocks of information are *symbols* and *meaning*, which cannot be reduced to one another. The *symbols of information* are the physical media of representation and the *means of transmission* of information. Without the associated meaning, the symbols of information have no significance since meaning is an ascribed and acquired quality and not an inherent property of the symbols. We can transmit symbols of information but cannot transmit meaning from one mind to another without a common protocol or convention. A concise and cohesive framework for information can be established on the common ground of the *mind*, *meaning*, and *symbols* trio. Using reasoned arguments, logical consistency, and conformity with common experiences and observations as the methodology, this paper offers valuable insights to facilitate clear understanding and unifies several definitions of information into one in a cohesive manner.

## 1. Introduction

We are constantly bombarded with vast amounts of sensory inputs through our five senses, thoughts, and emotions. We experience anything that enters our attention span with or without intention as *information*. Anything we observe or interact with physically or mentally can be a source of information. There are many *definitions* for information proposed in different contexts in diverse fields, such as linguistics, physics, computer science, information and communication technologies, statistics, thermodynamics, genetics, and philosophy.

Information can be defined in a broad sense as *something that informs*. That is, information is something with the *potential* to incite a sense of cognizance in a conscious mind, and in so doing, add to the knowledge of a conscious being. This commonsense depiction of information is consistent with the dictionary definition of the term and is in line with our intuitive understanding and everyday use.

Noting that one’s perception of being informed or the act of informing is a *mental phenomenon*, information is intimately connected with the *mind*. As such, the meaning component of information is *ontologically subjective*—it exists because of and relative to a conscious being. This is also the case for other subjective phenomena such as thoughts, emotions, pain, and pleasure since they all owe their existence to sentient, conscious living beings such as humans. The five senses such as sight, smell, and taste that are triggered by physical signals are actually mental renderings, too, since they are mentally constructed on the sensory electric signals that are transmitted to the brain by the nerves, in a display of how tangible and intangible entities interplay. Even *science*, which is epistemically objective, is ontologically subjective since there would be no such thing as science without conscious minds.

M. J. Bates [1] compiled representative definitions of information drawn from information science and related disciplines and compared them. She identified and described seven categories of definitions: communicatory or semiotic, activity-based (i.e., information as event), propositional, structural, social, multi-type, and deconstructionist. She also addressed the data–information–knowledge–wisdom continuum. As Bates correctly stated, there is no general agreement on what information is: “*it [information] is a term that has been defined in countless ways over many decades. It would be fair to say that there is no widely agreed-upon definition or theoretical conception of the term. The meaning of this term is still highly contested.*”

Some definitions of information are more confined and abstract than others. Information is said to be many things, such as knowledge, patterns with no inherent meaning, entropy, something that causes a change in the mental map, and the degree of uncertainty of a message, among other things [1,2,3]. Inconsistencies among different definitions of information are the norm rather than the exception, to the point that the term ‘information’ is often vague without specifying the related field. In this paper, we will use the term ‘information’ in the sense of something associated with *being informed* or *acquiring knowledge*, as stated above and discuss the fundamental attributes of it.

Some treat information as a *tangible* or *physical phenomenon*, while others treat it as something *subjective* or *intangible*. Though intangible in nature, information is not a disembodied abstract entity since it is always associated with tangible representation by physical symbols such as words, sounds, or the digital media of 0′s and 1′s. We adopt the familiar view that the meaning component of information is a subjective entity like knowledge, as discussed below, while recognizing the physical character of the symbols of information. This view is the most intuitive and plausible of all, and it fully conforms to our experiences and everyday use.

The pioneering work of B. C. Brookes [4,5] on the cognitive aspects of information science provided the philosophical and mathematical framework for quantifying knowledge as a *change of state of the mind* due to information input. The description of knowledge in this paper as a mental entity is consistent with Brooks’s description of knowledge as a change of state of the mind. Others such as A. Shaw [6], D. Bawden [7], L. Floridi [8], and C. Cole [9] have improved on this work and contributed to the progression of information science as they have quantitatively interrelated information with the modification of knowledge structure in the mind.

In a classic paper in information science, M. K. Buckland [10] identified three principal uses of the word ‘information’: 1. *Information-as-process*, which is the process of someone being informed by the act of informing via the communication of knowledge, 2. *Information-as-knowledge*, which denotes information that is perceived as ‘information-as-process,’ and 3. *Information-as-thing*, which refers to physical objects such as data and documents as they are regarded as being informative or as having the quality of imparting knowledge or communicating information. Buckland distinguishes between the last two types of information: “*A key characteristic of ‘information-as-knowledge’ is that it is intangible: one cannot touch it or measure it in any direct way. Knowledge, belief, and opinion are personal, subjective, and conceptual. Therefore, to communicate them, they have to be expressed, described, or represented in some physical way, as a signal, text, or communication. Any such expression, description, or representation would be ‘information-as-thing’.*”

In this article, we aim to complement the wealth of literature on the quantitative treatment of information by giving an overarching philosophical qualitative narrative of the concise conceptual structure of information and knowledge and the interrelations between various related concepts to depict an accurate integrated, holistic mental picture of information. For example, Buckland’s three meanings of information described above are preserved in the description presented here, but they are portrayed in a more intuitive and unified context as components of information: here, we refer to information-as-thing as the *symbols of information*, information-as-knowledge as the *meaning*, and the information-as-process as the *transmission of information*. Therefore, there seems to be a good correspondence between the relevant concepts used here and by Buckland.

We reserve the term ‘knowledge’ to represent ‘meaning’ that resides in a conscious mind, which is consistent with Buckland’s usage of the ‘information-as-knowledge’ term. This way, information is unified into one cohesive entity which fully conforms to our intuitive understanding.

It is hoped that the arguments presented here will provide a good grounding for the rightful places of mental phenomena such as *consciousness* and *meaning,* as well as the physical representations of *symbols* and *signals*. The conceptualizations presented are also hoped to clarify the widespread confusion regarding the nature of information, the symbols of representation, and the meaning.

We start by first developing a concise definition of information and describing the process of transmission of information. Then we establish the relation between information and knowledge, followed by describing the interrelation between knowledge and consciousness. In this regard, reference is made to the elusive mind and the central role it plays during the process. 

## 2. Information as Meaning Ascribed to Symbols

**Figure 1:**
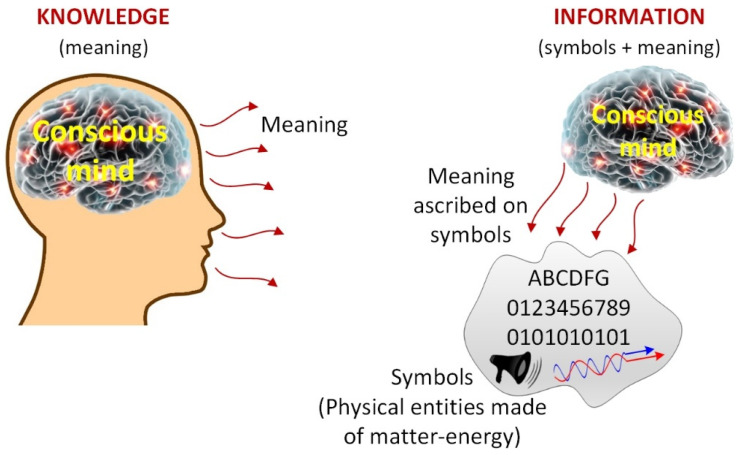
Information is meaning ascribed to tangible symbols by a conscious mind; knowledge is meaning that resides in the mind as percepts.

In its simplest form, all information can be reduced to a physical *symbol* and a nonphysical *meaning*. Symbols are physical entities since they are made of matter or energy, but this is not the case for meanings, which are mental entities. These two constituents are of different ontological natures and thus cannot be reduced to one another. Therefore, the two most essential ingredients of information are the *symbols* and the *meaning*—similar to syntax and semantics in languages. Symbols represent information, but they alone are not information. In the same way, *syntax* represents *semantics*, but it is not semantics—semantics resides in the mind. A tangible symbol of information always accompanies information, but the intangible meaning resides in a conscious mind, not on the symbol, as depicted in Figure 1. This is because *meaning is ascribed* to a symbol *externally* by a knowledgeable conscious mind as a posteriori.

The association of a symbol with meaning is *incidental*, not *intrinsic*. That is, symbols have no inherent meaning of their own. So, it is no accident that the meanings assigned to symbols are arbitrary, as evidenced by the same meaning assigned to different words in different languages. Different symbols can be used to represent the same meaning.

As a means of communication in a society, a *language* is an agreed-upon *convention* or *protocol* with fairly consistent correspondence between symbols and meanings such that a spoken or written word evokes essentially the same meaning(s) in the minds of all the speakers of that language. Symbols serve as the means of *representation* and *communication* of meanings and facilitate the transmission of meaning between minds.

Information is comprised of a symbol and meaning, but the principal ingredient is *meaning*, which is an invisible mental existence. That is, information is essentially a *mental construct*, just like the entire field of mathematics (or logic) and the laws of physics. As such, the existence of meaning is as real as mathematics and the laws of physics. Meaning is immaterial since it is not made of matter or energy. As such, meaning is ontologically subjective. Therefore, it cannot be perceived by the ordinary visual, auditory, tactile, olfactory, and taste senses. But as beings with conscious minds, we all have an innate sense and awareness of meaning, as we do other existence such as consciousness.

There is no such thing as *meaningless information*. Meaning is an essential property of information, even though it can be insignificant, vague, or just plain wrong. So, it is no surprise that when a text message can be interpreted differently, the correct interpretation is determined by contacting its author since the ascribed meaning resides in the author’s mind, not among the intricacies of the symbols. As cultural historian R. Tarnas [11] stated, “*Meaning is rendered by the mind and cannot be assumed to inhere in the object, in the world beyond the mind, for that world can never be contacted without having already been saturated by the mind’s own nature.*”

Symbols are physical states of assemblies of letters, numbers, signs (including traffic signs), marks, shapes, patterns, punched cards, emojis, sounds, waves, lights, motions, electric circuits, 0 s–1 s, DNA strands, etc. Bodily dispositions, known as body language, such as mimicry, gestures, behaviors, and vocalizations, are also used to symbolize information or convey messages. Even performing arts such as dance, music, and theatre and visual arts such as painting and sculpture can serve as symbols of information. In this case, artists assign meaning to the art to provide expressed and enacted information. But this may not be perceived by the general audience as information since there is not a common code of interpretation regarding arts.

The *sign language* that involves the shapes made by hands, lips, and the body also serves the same purpose for the hearing impaired. Although often vague, *body language* is an effective way of communication. *Recorded information* in books, drawings, and audio or video recordings is a major form of information for adults. But children learn primarily by observing and copying the *enacted information* in the real and virtual worlds.

The basic building blocks of languages are *words*, which are sequences of specifically shaped symbols called *letters* that constitute an alphabet, and the *sounds* associated with those words. In English, the sequence of the letters ‘a-p-p-l-e’ and the corresponding sound is agreed to represent the fruit we all are familiar with. So, when English-speaking people see or hear the word ‘apple,’ it evokes the same meaning in their minds, and they all visualize and understand the same fruit. The sentence ‘Apple is red’ lights up the same meaning in our minds and conveys information about the color of that fruit. Sometimes meaning varies with context, and the same symbols represent different meanings, as in the phrase ‘How r u? in fast texting.

In time, the mind tends to combine two things that always appear together into one, like combining the kernel and the husk into one. As a result, we *merge* the meaning ascribed to the symbols with the symbols themselves and view the sequence of the words ‘Apple is red’ as a meaningful sentence. This is especially the case when all the people assign the same meaning to the phrase and understand the same thing when they sense it. This conditioning leads the physical *symbols of information* to be viewed as the *information itself* since the meaning is identified with the phrase. The disappearance of the meaning when the symbols disappear reinforces this delusion.

If we ask ‘Does the sentence ‘Apple is red’ contain any information?’ we all will probably answer with an unreserved ‘Yes.’ We would even say that this is an informative statement. Probably we all have sent email messages stating that ‘the information you requested is in the enclosed document’ without giving it a second thought. This imprecise use of information is acceptable as long as it does not cause any misunderstanding. But when a discussion requires a distinction between the symbols and meaning for precision, we should ensure that we separate the husk from the kernel, and the symbol from the meaning.

Symbols are dull formations of matter or energy until someone attaches some meaning to them using a convention or protocol such as a language familiar to others. We hold that information is a mental entity or mental construct, as stated before. If there were no minds, there would be no information to speak of. Without minds, the physical universe with all its mass and energy would remain intact. But there would be no physical sciences such as physics, chemistry, and geology as branches of learning since there would be no experiencing sentient beings to generate and ascribe meaning. There would be no languages, either.

A pizza recipe in English, for example, means nothing and conveys no information to non-English-speaking people; it is simply an arrangement of words that are groups of letters. Only a person with knowledge of the English language can associate meaning with the words and sentences of the pizza recipe and see it as information. After all, learning a language is the process of matching symbols (like ‘Apple’) with conceptions (like the mental image of an apple); and matching the symbolic forms of sentences with the corresponding meaning in the mind. That is, linking *syntax* (symbols) with *semantics* (meanings). Again, symbols are easy to identify since they are visible and tangible. But the existence of meaning is often denied since meaning is invisible and intangible. Meanings are subjective entities and are outside direct scientific inquiry and scrutiny since they are not part of the physical realm.

Symbols may reside on various media such as *ancient tablets, papers, memory chips*, etc., but meaning resides only in minds, primarily in human minds. If all the humans on earth were somehow to lose their minds or disappear from the face of the planet, there would be no information to speak of in the world. There would still be piles of symbols of information but nothing meaningful since there would be no knowledgeable minds to ascribe meaning to those symbols. The computers would continue doing whatever they were doing without making any sense of their output until they ran out of power, and libraries would become repositories of well-ordered ink-tainted papers. The information that once manifested on those symbols would disappear, and the world would enter a new dark age. As science writer J. Horgan [12] put it: “*The concept of information makes no sense in the absence of something to be informed—that is, a conscious observer capable of choice, or free will … If all the humans in the world vanished tomorrow, all the information would vanish, too.*”

If human-like intelligent aliens were to come to the earth then and see the marvelous artifacts like buildings, cars, airplanes, TVs, smartphones, etc., and no humans around, they would deduce that once there were knowledgeable intelligent beings on earth since all those technological marvels can be built only with *intention, knowledge, skill, and ability*. But they would make no sense of the collections of symbols on paper or chips since they do not know the language they are written in.

There are diverse opinions about what constitutes information and whether it is a material or immaterial entity. Some take a physicalist stance and view information ontologically as physical or material since it is encoded in the materials, sound waves, electromagnetic waves, or the 0′s and 1′s of digital media that constitute its symbols. A typical example of this view is expressed by Bates as “*[T]he position taken here is fully materialist, that is, no abstract plane is assumed to house or manifest the information associated with the physical realities we experience. If the information is anywhere, it resides in the physical realities of nature, whether in the structure of a piece of granite, or in the neural pathways of the brain. To say this, however, is not to say that information is identical with the physical materials or waves that make up the pattern of organization. The information is the pattern of organization of the material, not the material itself*.” [13].

We do not subscribe to this view since the *patterns of organization of the material* constitute merely the *symbols of information*, not the information itself. Others view information as something abstract and immaterial. Information manifests itself in physical symbols, but it is not a physical entity. If it were, we could spread the information by making copies of the physical media that house it. We cannot increase the amount of information by making millions of copies of an information-laden book; doing that would only increase the accessibility of information. But we can increase knowledge by increasing the number of knowledgeable minds by encouraging people to acquire information from printed or electronic media. The fundamental forms of information are elaborated on by Bates [14]. We stress that the core of information is meaning, which clearly is not matter. As mathematician and philosopher N. Wiener [15] put it, “*Information is information, not matter or energy*.” Therefore, the arguments presented here are in line with those of Wiener, but not of Bates.

## 3. Information in the Field of Communication

The age we live in is called the *information age* or the *knowledge age*. Information and communication technologies (ICT) are reshaping all aspects of life. *Communication* is basically the transmission of information from one mind to another via any suitable means, such as written and spoken languages, sign language, body language, music, arts, and more recently, digital media and the internet. The definition of communication can be broadened to include smart devices since machine-to-machine, machine-to-human, and human-to-machine transmission of information is becoming commonplace in Industry 4.0. C. E. Shannon [16], known as the father of information theory, is rightfully credited for revolutionizing communication engineering with his pioneering work on information and the transmission of information by devising mechanisms to measure the amount of information conveyed.

**Figure 2:**
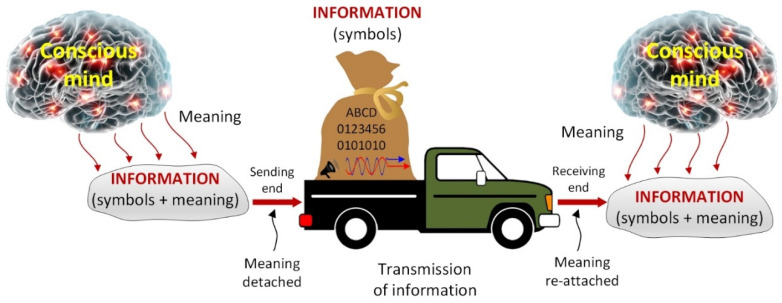
Transmission of information involves transmitting the symbols of information only; meaning is ascribed to the symbols at both ends of transmission by conscious minds.

All *means* of communication, including the air that allows sound waves to propagate, are physical. Therefore, communication naturally involves the transmission of the physical symbols of information, with the understanding that the transmitted symbols evoke the same meaning at both ends of transmission so that there is no loss of meaning, as depicted in Figure 2. That is, although the communication deals with the *transmission of symbols*, the associated meanings are also transmitted inadvertently with the symbols. The meaning detached at the sending end is reattached at the receiving end via the receiver’s mind where the meaning resides—even if the symbols are encrypted during transmission.

Therefore, in the technical field of communications, meaning is taken for granted, and information is treated as mere symbols. Then the priority becomes the accurate transmission of the symbols and the precise conveyance of the accompanying meaning by the transmitted symbols while making the process accurate, efficient, and effective. Although the transmission of information involves the transmission of symbols, the transmission of meaningless symbols is *not* transmission of information.

The so-called *transmitted information* is no information at all unless there are conscious beings at the receiving end who know how symbols correspond to meanings, and can ascribe the correct meanings to the symbols received. In the communications field, the phrase ‘*the transmission of information*’ means ‘*the transmission of symbols of information*.’ When *encryption* is used, the symbols of information are still transmitted to both authorized and unauthorized receivers. But the unauthorized ones receive no information since they do not have the protocol to *decode* the encoded symbols, and thus they cannot ascribe meaning to them.

Likewise, the meaningful messages sent to outer space intended for the *extraterrestrials* are meaningless symbols of information since there is no agreed-upon interstellar communication protocol. The electromagnetic signals that carry our messages will probably be treated as parasite noises by the receivers of the aliens unless they see patterns in those parasitic noises and attempt to match meanings to those signals by developing a code.

W. Weaver [17], one of the pioneers of modern communication theory, underlines this point: “*The word information, in this theory, is used in a special sense that must not be confused with its ordinary usage. In particular, information must not be confused with meaning. In fact, two messages, one of which is heavily loaded with meaning and the other of which is pure nonsense, can be exactly equivalent, from the present viewpoint, as regards information. It is this, undoubtedly, that Shannon means when he says that ‘the semantic aspects of communication are irrelevant to the engineering aspects.’ But this does not mean that the engineering aspects are necessarily irrelevant to the semantic aspects*”.

Weaver states that the concept of information developed in this theory at first seems disappointing because it has nothing to do with meaning. He then likens the communication theory to a discreet operator working at a now archaic *telegram station* transmitting messages. The operator pays no attention to the meaning, whether it be sad, joyous, or embarrassing. All she cares about is the fast and accurate transmission of the symbols that land on her desk. After all, once the symbols are transmitted, the meaning will take care of itself. The notion of information as meaning ascribed to symbols is consistent with both Shannon’s and Weaver’s depiction of information.

This discussion above on the transmission of information parallels M. J. Reddy’s *conduit metaphor* in linguistics [18]. It characterizes communication between people via a language that is viewed as a *conduit* that transmits mental content between people. In the conduit metaphor, speaking or writing is depicted as pouring *mental content* (meanings) into *containers* (words and sentences). Meanings are then extracted by listeners or readers.

Information and knowledge are often used interchangeably as synonyms in daily life. However, in information science, they are used as antonyms. In a counterintuitive sense, zero information means complete knowledge and thus zero ignorance, while maximum information corresponds to minimum knowledge, and thus maximum ignorance. As W. Weaver [17] put it, “*Information is, we must steadily remember, a measure of one’s freedom of choice in selecting a message. The greater this freedom of choice, and hence the greater the information, the greater is the uncertainty that the message actually selected is some particular one. Thus, greater freedom of choice, greater uncertainty, greater information go hand in hand*”.

When a piece of paper with printed information is put into a blender with water and the blender is turned on, the ‘print’ is gone forever. But nothing happens to information as long as it exists in at least one conscious mind, on another paper, or a website on the internet. When a fire breaks out in a library, it destroys the symbols of information in the books, but not the information itself—especially in this internet age. If the burned books have print or electronic copies at other places, no information is destroyed due to this fire [19].

When information is transmitted on the web, web browsers simply reproduce the symbols on the screen by converting electrical energy to light energy at the tiny pixels on the screen instead of transmitting the physical symbols themselves. That is, what is transmitted on the internet is merely syntactical instructions related to the reconstruction of symbols representing information. Fax machines do the same thing, except they form the symbols of information on sheets of paper with ink at the receiving end rather than turning the pixels on and off.

## 4. Information and Knowledge

Information and knowledge are closely related, but there are significant subtle differences. The essence of both knowledge and information is ‘meaning,’ as stated before. *Information* is meaning represented by physical symbols such as sights, sounds, and words which can be perceived by all via the five senses. *Knowledge*, on the other hand, is meaning that resides in the mind, and thus there can be no knowledge without the mind. Knowledge is intimately associated with a mind that knows; and knows that it knows. As such, entities with no conscious mind—like robots, smartphones, and plants—do not and cannot have any knowledge.

Symbols constitute an essential part of information, but not of knowledge. Information is like a glittering fabric woven out of the threads of meaning and symbols. Knowledge cannot be perceived or retrieved by outsiders, but information can. In information, meaning is ascribed or imparted by a conscious mind to a physical symbol. Therefore, *knowledge becomes information when it is represented by a symbol that is comprehensible to others*. Knowledge is internal, but information is external. The meaning in information is like a spirit that transcends a physical body composed of the symbol. That is,

Information = Meaning ascribed to symbols (external)

Knowledge = Meaning that resides in a conscious mind (internal or mental)

This description is consistent with the elegant definition of information as “*physical surrogate of knowledge*” by J. Farradane [20], credited for coining the phrase ‘Information Science.’

It can be said that the difference between *information* and *knowledge* is the *person* as the beholder of knowledge. Although information and knowledge are essentially the same and often used interchangeably, information is ‘out there’ on symbols, but knowledge is ‘in here’ in the subjective mind. Knowledge is internal and hidden; information is external and in the open. Information can be accessed by others via the search engines like Google, but knowledge cannot. We can say that knowledge is acquired information that is internalized and integrated with the knowledge base of a person. Newly acquired information by a person becomes new knowledge and raises the level of knowledge of that person. Commonly known information is also referred to as *common knowledge* since it resides in many minds. In some languages, there is only one word for both information and knowledge. In such cases, qualifiers like ‘in the open,’ ‘out there,’ and ‘in the mind’ can be used to distinguish the two.

It appears that knowledge affects the state of a mind and the world image of a person. The input of information changes in some way the knowledge structure of the mind. Popular author and philosopher K. E. Boulding [21] used the concept of the ‘image’ as the grand total of one’s subjective knowledge, or the mental image of the world, and their place in it. A. D. Madden [22] defined information as something that alters this image: “*a stimulus which expands or amends the World View of the informed*.” A. D. Pratt [2] expands the same concept by spelling information as ‘in-formation’ and stating, “*After a person has received and understood the content of a message, in ordinary speech we say that he has become informed about the matter at hand. This is a surprisingly precise and accurate statement. He has been ‘in-formed.’ … He has been inwardly shaped or formed; his Image has been altered or affected. In-formation is the alteration of the Image which occurs when it receives a message*.” In the same line of thought, B. C. Brookes [5] expressed this concept mathematically by an equation between information *I* and knowledge *K* as *K*[*S*] + Δ*I* = *K*[*S* + Δ*S*], which indicates that the knowledge structure *K*[*S*] is changed to the new modified structure *K*[*S* + Δ*S*] by the information input Δ*I*. In the end, what matters the most is the cognitive maps or pictures of the individuals.

Philosopher F. I. Dretske [3] describes information as the ‘*commodity capable of yielding knowledge*.’ For example, a detailed account of the photosynthesis process exists in books, videos, websites, and the minds of many. Therefore, photosynthesis exists as information, even if I have no knowledge. In this case, photosynthesis is knowledge for many, but not for me. Your knowledge of photosynthesis acquired by reading a book is information for me since it exists out there represented by symbols, but not in my mind. When you tell me about photosynthesis, you are loading your knowledge on spoken words as the vehicle of transmission, and conveying the information to me via the sound waves that are perceived by my sense of hearing. The knowledge in you becomes information out there when it leaves your mind via your written or spoken words and gains permanence when it is recorded. When the information reaches my mind through my eyes or ears and is internalized, it becomes part of my knowledge base. When I forget all about photosynthesis, my knowledge of it is gone; but nothing happens to the information about it out there represented by symbols.

The act of *reading a book* is the process of *transmitting the information* in the book to the mind of the reader through the eye and converting the information into knowledge once it is internalized and integrated. The acts of reading, listening, and watching are acts of feeding information into the mind for consideration and integrating with the knowledge base if endorsed by the mind. Depending on how they interpret the information there, different people reading the same paragraph may understand different things. After all, we are perceiving what our biased mind is telling us. As A. Seth [23] put it: “*We don’t just passively perceive the world, we actively generate it. The world we experience comes as much, if not more, from the inside out as from the outside in*”.

Consequently, we all sense the same physical world, but each person mentally constructs and lives in a different virtual world. In the end, what we perceive as reality is what the mind makes out of the inputs of sensory signals from the outer physical world, the past experiences stored in memory, and the innate perception of thoughts, emotions, and intuitions. So, it is no surprise that two people looking at the same thing or event do not necessarily perceive the same thing because of the differences in their conditioning from past experiences and their present mental state.

What we call *perception* is perhaps the most disguised deception since perception is a *virtual reality simulation* of reality reconstructed by our minds, not the actual external reality itself. Our knowledge of the physical world is derived from our perceptions manipulated by the mind to give us the impression of reality. Moreover, the perceptual experience is fallible since the world is not always as it appears to us [24].

As physical entities, *symbols* are inherently meaningless by their very nature, as stated before, unless there are known meanings assigned to them by a conscious mind—like the meanings attached to certain sounds in spoken languages. Symbols serve as the *signs, flags, tags, prompts, triggers, labels, marks*, or *markers* associated with meaning by a protocol, contract, convention, or agreement. They represent information, and thus they are ‘*meaning holders*.’ Symbols are also used to store information by registering them on rocks, paper, magnetic tapes, memory chips, or other media using a suitable recording technology. Symbols can be perceived directly by the five senses or indirectly by electronic gadgets and transmitted to the mind as electric signals that are decoded and interpreted again by the mind.

Without meaning, we merely have *clusters of symbols* that represent no information, and thus mean nothing. Without symbols, on the other hand, we cannot perceive the information through our five sensory organs, which act as our biological sensors of the physical realm. Therefore, we can say that there would be no information out there without the symbols. There would just be knowledge in individual minds, which could not be transmitted from one mind to another without symbols. This interdependence of the symbols and meaning of information, and the fact that information cannot exist without symbols, has deluded many to *misconstrue symbols as the information itself*. To avoid such misunderstandings, we must separate information into its two main components of *meaning* (the intangible core or kernel) and *symbol* (the tangible shell) whenever necessary for clarity. Although the meaning and the symbols of information are intertwined, information cannot be reduced to its symbols.

Thanks to the *search engines* in computers and smart devices, we are as close to information as our fingertips, regardless of where the digitized information actually resides. Information *originates* from the knowledge in one mind and eventually ends up as knowledge in another. Physical symbols are vehicles for the *transmission of information* from one mind to another. Information that manifests on symbols of physical media is a reflection or association phenomenon since the essence ‘meaning’ cannot be found in the basic building blocks of the physical symbols by a reductionist approach.

Information can be said to be *implicit* or *hidden* before it is known by anybody, and *explicit* or *open* after it is discovered by at least one person. Obviously, photosynthesis was commonly occurring on earth before any conscious being was aware of it (information in use). It became *open information* after someone discovered photosynthesis and wrote about it (information in print). It has become *common knowledge* as several people learned about it.

Raw bits of disorganized facts, figures, values, recordings, or entries gathered for the purpose of processing, interpreting, and making sense of them are usually referred to as *data*. A pile of data, such as an array of numbers or a cluster of charts, is not information. Organized, processed, contextualized, and interpreted data become information, which turns into knowledge when perceived and processed by a conscious mind, and is integrated with a person’s knowledge base. Repositories of information about people, goods, or processes for use by governments, businesses, or other organizations, especially in digital media, are called *databases.* Information is also defined by Glattfelder [25] in terms of data as ‘*a construct that consists of one or more well-formed and meaningful data*’.

In *philosophy*, the necessary and sufficient condition for a proposition to qualify as *knowledge* is for it to be justified, true belief. That is, for a proposition to constitute knowledge, (1) it must be true, (2) it must be justified, and (3) the proposer must believe in it. Therefore, knowledge is usually expressed as ‘*justified true belief*.’ Knowledge in this sense constitutes *confirmed knowledge*. Most epistemologists maintain that what is false cannot be known. Also, failing to believe something precludes knowing it. You can only know what you believe. The justification condition limits knowledge to propositions that are epistemologically proper. As such, opinions are insufficient to qualify as knowledge even if they are true. But all these conditions are controversial and are still debated [26].

There exists extensive literature on justified true belief, such as by Parikh and Renero [27], that treats knowledge from a philosophical perspective. Here we use the phrase *knowledge* in a broad sense, as elaborated above, rather than a restricted philosophical sense of justified true belief. Budd [28] examines the relationships between meaning and truth as they may contribute to a constitutive definition of information. He posits that information can only be defined within the context of meaning and truth and attempts to integrate meaning and truth in a new way.

## 5. Knowledge and Consciousness

Consciousness can be viewed as the individual *awareness* of thoughts, memories, senses, the environment, and even the sense of awareness itself. Knowledge and consciousness are closely related since both are associated with the awareness of something physical or phenomenological. Consciousness is a *mental state* directed at something with the intention of *being aware of* or *having knowledge of* that thing. We are conscious of even our own consciousness since consciousness *transcends* itself. We cannot claim knowledge of anything unless we are conscious or aware of the phenomenon of knowing. The mechanism of consciousness kicks in when we wake up in the morning, and goes on until we fall asleep, faint, or enter a state of coma.

Without consciousness, there would be no awareness of existence, and thus there would be no existence to speak of. Therefore, it can be said that consciousness represents the mode of existence of both tangible and intangible entities. We are conscious of even our dreams. Otherwise, we wouldn’t be aware of the existence of dreams. It seems that a person constructs a projected virtual world organized around himself—a world of representations brought into existence by consciousness.

It is through consciousness that we can attribute meaning to percepts. Therefore, at the core of the phenomenon of knowledge lies consciousness, which is the enigmatic sense of awareness of one’s innate states. From pots and pans to smartphones, everything made with information is associated with conscious beings, since meaning, which is the kernel of information, resides in the minds of conscious beings. Even in *archeological excavations,* this understanding prevails as a key criterion: randomly shaped stones are discarded as arbitrary acts of nature. But unearthed *sculptures, cylindrical marvel columns, metal disks with engraved inscriptions,* or even *needles* are cherished as archeological artifacts since they can only be made by knowledgeable conscious beings with intention.

Therefore, from an ontological perspective, it can be said that the *common thread* of all meaningful existence is information. The *primal ingredient* of all meaningful human-made things is also information. The process of making physical items such as pots, cars, and phones is essentially the *embodiment* of mental abstractions that are weaved out of the threads of information. The informational abstractions are called *designs* and are represented physically as blueprints. The process of 3D printing, also called *additive manufacturing*, is merely the manipulation and arrangement of matter as stipulated by the information encased in the software.

We have knowledge of everything that we are conscious of at this moment. But we are not conscious of everything that we know since our knowledge base extends beyond the current moment. It includes prior knowledge stored in our *memory* that we access as needed. Therefore, in conscious beings like humans and higher animals, memory represents retrievable passive knowledge. The *subconscious realm* represents knowledge that somehow exists in the mind of a person, but is not readily accessible to the consciousness. As such, subconscious existence is outside the sphere of awareness. Yet, it can be accessed by the waning of the physical perception and induction of an altered state of consciousness through suggestion, meditation, hypnoses, dreams, and sometimes trauma.

The *phenomenon of knowing* is directly associated with consciousness or awareness. Then it can be posited that unconscious entities like *computers, smart devices, and robots* can have information (actually, symbols of information), but not knowledge since they are not aware of that information and cannot experience it. We as humans are aware of the information we have and we sense it. Memory for such devices represents retrievable information stored on chips.

With the advent of computing machines in the 1940s, the prospect of machines having human-like attributes such as *thinking*, *understanding*, and *consciousness* has been hotly debated. In 1950, the *Turing test* was proposed by Alan Turing to assess whether machines can think [29]. The test involves a human questioner interrogating a *human* and a *machine* in writing in a natural language. If the questioner cannot tell the machine from the human based on their responses, the machine would be said to have passed the test and judged to be a *thinking* or *intelligent* machine.

In 1972, Hubert Dreyfus critiqued the common questionable assumptions regarding artificial intelligence (AI), such as the *human brain* resembling a *digital computer* and that understanding can be codified [30]. Later, Roger Schank and other AI researchers claimed that their AI programs could literally *understand* English sentences [31].

This prompted renowned philosopher John Searle to challenge this notion in an article pivoted around a simple yet powerful *Chinese Room analogy* or *argument* [32]. It has become one of the best-known thought experiments in the philosophy of mind, language, cognitive science, and consciousness regarding AI. He re-described his Chinese Room Argument as: “*Imagine a native English speaker who knows no Chinese locked in a room full of boxes of Chinese symbols (a database) together with a book of instructions for manipulating the symbols (the program). Imagine that people outside the room send in other Chinese symbols which, unknown to the person in the room, are questions in Chinese (the input). And imagine that by following the instructions in the program the man in the room is able to pass out Chinese symbols which are correct answers to the questions (the output). The program enables the person in the room to pass the Turing Test for understanding Chinese but he does not understand a word of Chinese*.” [33].

Searle then made the point that if the man in the room does not understand Chinese on the basis of implementing the appropriate program for understanding Chinese, then neither does any other digital computer on the same basis.

Computer programs are just *syntactical*. They only act on the physical symbols, with no regard for their *meaning*. Computers *manipulate* symbols per encoded instructions in the program, but manipulating symbol strings using syntactical rules does not generate *semantics* or *meaning*, thus a sense of *understanding*.

The sense of understanding is closely related to the sense of *awareness* and thus *consciousness*. Searle extended the Chinese Room Argument in his follow-up articles to demonstrate the close connection between *understanding* and *consciousness*. He also argues against the computationalist notion that the *mind* is an *information-processing system*, just like a computer. He asserts that computers at best can *simulate* the human mind, but simulation is not *replication* or *duplication*: “*Computation is defined purely formally or syntactically, whereas minds have actual mental or semantic contents, and we cannot get from syntactical to the semantic just by having the syntactical operations and nothing else. … A system, me, for example, would not acquire an understanding of Chinese just by going through the steps of a computer program that simulated the behavior of a Chinese speaker*” [34].

Searle stands firm on this position since it is well-known that the computational processing of computers and robots is solely *syntactic*, and computational processes and their outputs exist without a *cognitive state*. Sometimes there appears to be a *distinction* without an apparent *difference*—like a humanoid or zombie that looks and acts like a human, yet lacks consciousness. A computer may beat a human in a chess or go game, but it has no idea of what it is doing. Searle underscores that *computer understanding* is not just partial or incomplete; it is *zero*. David Cole gives a comprehensive account of the Chinese Room Argument and several counter-arguments [35].

Unlike computers, minds have *mental contents* like meanings, and associate meanings with *physical symbols*. Symbols such as words gain significance from the meanings they evoke in the mind and the emotions and understanding they induce. Computers manipulate syntax and produce appropriate syntactical output. But *syntax* does not produce *semantics*; thus, computation cannot produce *mental content* or minds [36].

Although some theories of mind hold that *mind* and *cognition* are *computational*, Searle maintains that we cannot get semantics from syntax alone and that semantics for a symbol must be provided separately: “*Formal symbols by themselves can never be enough for mental contents, because the symbols, by definition, have no meaning (or interpretation, or semantics) except insofar as someone outside the system gives it to them*” [37].

Smart devices are typically loaded with information, but they don’t qualify as *knowledgeable beings* since they do not possess consciousness. Microprocessors and information transmission devices are purely *syntactical devices*. Their operations are defined syntactically with no semantics involved. *As discussed above, syntax is not semantics, and we cannot get semantics from syntax*. Likewise, we cannot get a cognitive experience such as *understanding* from the *intense signal processing* in a microprocessor. After all, computation is *symbol manipulation*—usually the manipulation of 0′s and 1′s or the electric signals in the transistors—which is purely *syntactical* and void of any semantics. *Meaning, semantics,* and *understanding* are phenomena associated with the subjective conscious *mind*. We contend that without consciousness, a machine cannot cross the gap between the *syntax of the symbols* to the *semantics of the understanding*.

That said, we commonly use phrases like a smartphone *knowing Spanish*, or an autopilot knowing how to fly an airplane or drive a car. This is because we often use the terms information and knowledge interchangeably in daily life. The knowledge we speak of here exists in the minds of conscious observers, not in the microprocessors of the devices. That is, the knowledge associated with smart devices are *observer-relative* phenomena. If we humans were suddenly to disappear, all those marvelous intelligent machines would turn into inept dummies.

Artificial intelligence is essentially *software*. It consists of instructions coded in 0′s and 1′s. The AI expert C. Cole [9] advocates the consciousness approach to information rather than the computational approach, which is the view that AI can imitate human thinking. He distinguishes the learning of humans and machines as follows: “*At the moment, AI machines can learn on their own, going beyond their instructions, but only in limited realms with controlled databases (Watson playing chess or the game Jeopardy). They can “learn” by detecting new patterns in the outside data environment, and categorizing the patterns into concepts, which would constitute a new response framework beyond what their original programmers’ envisaged. But this is not how humans produce new knowledge. Learning new concept categories is one step, and not the most important step in new knowledge production. We humans have another, deeper way of thinking about the world than simply detecting, categorizing, and conceptualizing patterns in the data we find around us in the outside world. New knowledge production is started by something else, something deeply rooted in our consciousness*.” The mathematical physicist R. Penrose [38] also contends that some aspects of the human mind lie beyond computation, and maintains that there is ‘*something*’ in the conscious activity that transcends computation.

As the current state of awareness, consciousness encompasses things that we are currently experiencing either *physically* via the five senses, *mentally* via thinking, or *emotionally* via feeling. The objects of awareness may include thoughts, dreams, imaginations, inspirations, reactivated memories, and physical entities. When we faint, we are unaware and thus unconscious and unknowing of anything. There is no perception when a being is unconscious. *Cognition* is also closely related to knowledge since conscious cognitive mental activities such as *thinking, reasoning, understanding,* and *remembering* can result in the discovery of new phenomena and the generation of new knowledge by conscious beings.

We become conscious of a percept, a thought, a volition, an emotion, or anything else when we direct our attention toward it. The *sense of consciousness* is the momentary *active* or *experienced* knowledge, which is the projection of one’s knowledge base on the *current moment*. Active knowledge involves attention. We all know that we have lungs. But it becomes active knowledge only when we direct attention to the lungs.

Memory is the passive knowledge that can be reactivated at will. Memory serves as the *repository* of personal information in one’s mind. The things we experienced a moment ago have now become part of our memory. Items recorded in our memory in the past but are erased now are no longer part of our knowledge. The phrase ‘I don’t know’ or ‘I don’t have any knowledge of it’ simply means ‘I am not aware of it’ or ‘I have no memory or recollection of it.’ Therefore, knowledge can be viewed as the combination of consciousness and memory:

Knowledge = Consciousness (active knowledge) + Memory (passive knowledge)

Without consciousness, there can be no ‘knowing’ or ‘active knowledge’. An unconscious person (in deep sleep, coma, or seizure) is an unknowing person as long as he or she remains in a state of unconsciousness.

Like consciousness, the notion of *knowing* is also a sensation, a feeling, and an experience. Therefore, only sentient beings such as humans and higher animals can have a sense of knowing. The *capacity* of a human being or an animal to acquire knowledge is proportional to the capacity of their consciousness. The more things a living being experiences and thus is aware of (physical entities, acts, thoughts, emotions, etc.), the higher the capacity of that being to learn.

Although they are alive and exhibit sophisticated cognitive capabilities, *plants* cannot have knowledge since it is generally accepted that they do not have consciousness. The humble leaf, for example, is the site of conversion of sunlight and carbon dioxide into chemical energy through photosynthesis. The *leaf* resembles a modern elegant *chemical factory* with no noise and waste. As such, it exhibits a wealth of information in action to observers. But a leaf does not possess any knowledge, because it is unaware of what it is doing. In other words, a leaf (and the entire plant, for that matter) is not a *knowledgeable being*.

There seems to be considerable *information processing* in the plants, like the weaving of the leaves and undertaking the photosynthesis processes. But there is nothing within the plants—not even a central nervous system—that is aware of what is going on. Therefore, it seems that any consciousness associated with the plants resides in the minds of external intelligent observers like us, not the plants themselves. That is, the apparent knowledge in the plants is not intrinsic; it is observer-relative. There is considerable debate on plant consciousness [39,40]. Here we take the position that, as an insentient entity, a plant has *zero consciousness* and thus *zero knowledge*.

*Higher animals*, on the other hand, are generally thought to be conscious of the leaves, and they acquire and retain knowledge about where the most abundant leaves are for grazing and when. Humans, as beings of the highest degree of consciousness, go well beyond and acquire knowledge about the intricate chemical processes occurring in the leaves. Armed with that knowledge, they go on to build artificial leaves that convert sunlight into fuel while removing harmful greenhouse gases from the atmosphere.

If *humans* remained *hunter-gatherers*, competing with other animals for food and shelter, there would be no difference between humans and animals. They all would have just adequate consciousness and knowledge to notice and deplete the resources in their environment to preserve their livelihood. What sets humans apart from other species on earth is their *high degree of consciousness* and their high potential to *acquire knowledge* that exceeds the visible realm. That is, to go beyond the visible physical world and delve into invisible mental arenas with subjective percepts. That *potentiality* comes with a unique mind equipped with the *intrinsic ability* for *high awareness, vivid imagination, curiosity, deep thinking, comprehensive understanding*, and the *joy of learning*. These distinctive attributes set humans apart from all other species, regardless of the degree of genetic similarity. *Curiosity*, for example, sparks inspiration, and inspiration drives innovation.

Through reflective thinking we can deduce that there are *degrees of knowledge*, depending on the level of consciousness involved, starting with the unintentional *casual awareness* of the environment. There is no intent involved in casual awareness since it is not a purposeful act, and thus involves no directed attention. Knowledge that arises from such unintentional experiences is stored in the temporary memory, to be disposed of after a while if it fails to attract the attention of the intellect for further inquiry.

Consciousness is closely associated with the innate qualities of attention and intention, and there are several theories associated with each [41,42,43]. The mind’s capacity to consciously entertain several trains of thought simultaneously is limited, forcing it to be selective. The process of selectively directing consciousness to a certain thought or item and focusing awareness on it is known as *attention*. Of course, this mental process involves the weighing of the sensory information received and the priority assigned to it. *Intention* differs from attention in that intention is a purposive voluntary act that involves a will. There is a primary reason or motive behind intention. It is the association with a primary reason that makes an act count as intentional. Both attention and intention can be shifted from one thought or item to another. And attention can even be directed to intention.

Observations on the variations in the intensity of the electrical activity in the brain during storing and retrieving information show that different parts of the brain are associated with different types of experiences. *Explicit memories* about our experiences and general knowledge base are related to the hippocampus, the neocortex, and the amygdala while *implicit memories* such as motor skills are related to the cerebellum. *Short-term working memory* is associated mainly with brain activity in the prefrontal cortex [44,45].

The *level of knowledge* is proportional to the *level of interest and attention* of the learner, and thus the level of consciousness involved. Knowledge is said to be at a high level when acquired after a meticulous investigation. When visiting a zoo, for example, we can acquire a high level of knowledge about the animals we are most interested in while barely noticing other visitors, vegetation, or the little reptiles running around.

In an attempt to explain embodied perception-action loops in neuroscience, renowned neuroscientist Karl Friston introduced the *free energy principle*, also known as *active inference*, which describes the representational capabilities of physical systems in biophysics and cognitive science. It postulates that the dynamics of physical systems minimize free energy, which is related to the negative log probability of some outcomes and resembles Shanon’s definition of information and thus entropy. In his own words, “*perceptual processes are just one aspect of emergent behaviours of systems that conform to a free energy principle. The free energy considered here measures the difference between the probability distribution of environmental quantities that act on the system and an arbitrary distribution encoded by its configuration. The system can minimise free energy by changing its configuration to affect the way it samples the environment or change the distribution it encodes. These changes correspond to action and perception respectively and lead to an adaptive exchange with the environment that is characteristic of biological systems*” [46].

Friston views the brain as an *inference engine* and assumes free energy to be the underlying principle of all biological reactions. He attempts to establish correlations between brain activity and perceptions. This paper takes the mind and consciousness as given, and maintains that meaning is not an emergent property of the symbols of information.

## 6. Intuition and Introspection as Sources of Subjective Information

*Perception* is the innate *sense of awareness* produced in us. It is called *physical* (or *sensible*) *perception* if the sense of perception is received through the five senses and thus originates from the physical realm. The innate sense of perception not associated with the five senses, such as thoughts, emotions, excitement, enthusiasm, and initiation, is called *intuition* or *introspection*. *Experience* is the awareness of the signals of both sensible perception and intuited perception, which constitutes the raw material of knowledge. Experience is the underlying phenomenon of both consciousness and knowledge. *Knowledge* starts with the perception of the signals that stem from the physical, mental, or emotional realms. It ends in the mind by processing and interpreting those incoming signals.

Once we consider information to be either *epistemically objective* (observer-independent) or *epistemically subjective* (observer-relative), there seem to be three routes of accessing information and thus acquiring knowledge: (1) the well-known *sense perception* of the outer world via the five senses by turning attention outwards, (2) *introspection* via turning the mental eye inwards, and (3) *intuition* via flashes of innate perception. Of these, *sense perception* is epistemically objective and thus observer-independent since it can also be experienced by others and be confirmed independently. As such, the outward sense perception establishes the common reality. *Introspection* and *intuition* are epistemically subjective and thus observer-relative since they exist in the eye of the beholder and cannot be confirmed by others. We can gain valuable knowledge by turning our attention inwards to observe our thoughts and feelings.

The terms *intuition* and *introspection* are related to innate cognition and knowledge. Introspection is associated with inward-looking into one’s own mind to examine one’s own *thoughts and feelings*. Knowledge gained by introspection is called *introspective knowledge*. Intuition, however, is associated with sudden insight into something such as innate perception of knowledge, instant cognition, immediate awareness, and flashes of creative insight. Knowledge gained by intuition is called *intuitive knowledge*, and it plays an important role even in scientific discoveries. Many intellectual breakthroughs have come about in a *flash of intuition*. Information transmitted by brain waves does not fall into this category since the brain waves are physical entities with certain physical properties. Thus they qualify as legitimate symbols of information. Introspection and intuition are the phenomena of attaining direct knowledge or cognition without involving physical perception and without evident rational thought and inference.

The modern practice of *psychology*, for example, relies heavily on *introspective knowledge*—knowledge attained by looking inward to examine one’s own thoughts and feelings—as reported by the clients regarding their inner *affective, volitional* and *cognitive states* without evident rational process. This practice reflects the presumption that consciousness has introspective access to the inner states of the mind. Clinical psychologists probe the minds of their clients by asking them to *observe their thoughts and emotions*, making introspection an indispensable diagnostic tool. Actually, scientific findings are the outcome of the scientists’ observations of their own subjective mental states and thus are based on introspection.

Before the recent qualitative turn in psychology, *behaviorists* would limit knowledge to the epistemically objective type such as those obtained by observing behavior and physiological measurements [47]. But with the advent of *cognitive science*, epistemically subjective knowledge as obtained by introspection via examining one’s own thoughts and feelings through interview methods became just as credible and reliable [48,49]. The qualitative methods in *social science* rely on introspection on the parts of both the participants and the researchers while exploring the individually constructed subjective worlds, with no claim to depict an objective reality. The basic pillar of such methods is the *irreducibility* of subjective experience to objective ontology.

We tend to look down on experiences that are not connected with physical perception. But intuition conceived in the mind, such as the experiences of *sudden insight* that strikes out of nowhere, can be as powerful. The French philosopher Rene Descartes’s famous quote “*I think, therefore I am*” associates *intuitive awareness of thinking* with existence. A thinking person knows with absolute certainty that there is thinking going on. There can be no mistake that there is an *entity* that does the thinking, and that entity is me. When I become aware that I am thinking, it is self-evident that I exist—regardless of the truth or falsity of the contents of the thoughts. We *intuitively know* that it is impossible to think without existing. Therefore, intuition and introspection should be regarded as credible sources of information, even though they do not strike us as being as concrete as sensory information that stems from physical perceptual inputs.

As the philosopher B. Longuenesse [50] points out, “the truth of ‘I exist’ is a necessary condition of the truth of ‘I think’; and that ‘I think’ is both true of the current thinker of the proposition and known to be true by the thinker of that very proposition, just by virtue of her thinking the proposition.” She also lays down the conditions under which perceptual inputs can be recognized as meaningful information—specifically, combining the inputs into a mental image, comparing the image to a concept, and then reflecting:

“*For perceptual representations to be ‘something to me’ is for them to be recognized under a concept, for instance ‘tree,’ which means that I can come up with the judgment ‘this is a tree.’ But I am able to come up with such a judgment only if I have bound (‘synthesized’) a variety of perceptual inputs and compared the resulting bundles in such a way that I can come up with a concept, in this case, ‘tree.’ The fact that my statement ‘this is a tree’ is backed by such a process of combining, comparing, and reflecting is what I am expressing when I say ‘I think this is a tree,’ thereby indicating that I am in a position to provide justification for my judgment. Unless I had been through the process of combining, comparing, and reflecting that makes it possible for me to accompany my representations with the thought ‘I think,’ concepts would be impossible and intuitions would be nothing to me (they would not be recognizable as representations of something, e.g., a tree)*”.

[50]

## 7. The ‘Symbol-Meaning-Mind’ Trio

All symbols of information are lifeless physical entities made of matter or energy, and all *clusters of matter and energy* are intrinsically meaningless. Any suggestion to the contrary is implausible. Reorganizing matter into a particular shape (such as dispersing some ink from a pen to form the sequence of the letters A-P-P-L-E on a paper) does not bestow meaning on it. The pattern of ink in the inscription APPLE is meaningless, except for those whose minds are equipped with the knowledge of the English language. This is similar to reorganizing matter in the form of a living being and expecting the organization to bestow life on it. It has never happened, and there is no indication to give us hope that it ever will. Assemblies of matter incite the emergence of *properties* that *passively qualify* the assembly, but not the emergence of *agencies* with causal power that subjugate and *actively govern* the assembly, such as life [51].

For example, philosopher E. F. Schumacher [52] explains the implausibility of the notion of reducing life to matter with a metaphor as follows: “*To say that life is nothing but a property of certain peculiar combination of atoms is like saying that Shakespeare’s Hamlet is nothing but a property of a peculiar combination of letters. The truth is that the peculiar combination of letters is nothing but a property of Shakespeare’s Hamlet. The French or German versions of the play “own” different combinations of letters*.”

This metaphor equally applies to reducing meaning to matter or energy. Considering that meaning can only exist in the minds of conscious living beings, the claim that the symbols of information *intrinsically have meaning* is equivalent to the notion that the symbols of information *have a mind*, and thus symbols such as words are conscious living entities.

Noting that *meaning resides in a mind* and the meaning of information is extracted from a mind through association, it is logical to say that *no information will exist without a mind*. Dictionaries, for example, would be meaningless and worthless piles of inked paper without a conscious mind. Therefore, the mind must be instituted as the *necessary external agency* of information. Then any discussion on information should revolve around the ‘*symbol-meaning-mind*’ trio. After all, it is the mind that first gives meaning to symbols, and then conjures meaning from the symbols of information to induce learning and understanding. Even the symbols used to represent information as well as the protocol or correspondence between symbols and meaning are the creations of the minds of conscious beings. Again, we stress that the *symbolic representation of information* should not be confused with the *information itself*.

Information exists only when the *symbols of information interact with a conscious mind*. That is, no information exists if *no interaction* is taking place with a conscious observer. The symbols of information on this page (the sequence of letters and words) are information only because our conscious mind interacts with them.

The *mind* is the subjective entity that *transmutes symbols of information into meaningful entities*. With the innate ability for extrasensory perception such as instinct, intuition, inspiration, and imagination, the transmuted world of the mind goes beyond the perceptions that stem from the world of symbols. As such, the mind is greatly enriched and gains significance from these multifaceted experiences which become a source of awe and amusement. If the mind is taken out of the picture, meaningful information reduces back to meaningless assemblies of symbols.

The notion of mind is hotly debated among philosophers and neuroscientists as the centuries-old *mind-body problem*. We will not get into the discussions of whether the mind is an illusion, a creation of the brain, or a separate entity distinct from the body. In any case, the elusive *mind* is the name used for the supposed center of *perception, experience, consciousness, imagination* and *knowledge*. There can be no perception or experience without the mind. It is the faculty of conceptualization of experiences and making sense of them.

*Intellect* is the aspect of the mind that deals with *reasoning, thinking, judging, finding causal links, noticing similarities, recognizing patterns, forming associations between related phenomena*, and *inferring via deduction or induction*. As such, intellect is a source of original information. Meanings that appeal to reason, logic, analysis, evaluation, and judgment are somehow routed to the intellect. *Imagination* is the faculty of the mind that depicts mental images for both tangible and intangible entities.

The human mind appears to be able to *encode* aspects of the physical world as abstract representations and record them, which is the act of *knowledge generation*. It also can *decode* them back into a physical form, which is an act of *expression*. The mind mediates between the physical and abstract realms of existence during knowledge acquisition and use. Knowledge is derived from sensory input, but apparently, it is constructed by an enigmatic personalized mental cognitive mechanism.

The *mind* itself and its numerous aspects or faculties are all mental entities, and thus of subjective nature. Despite their different roles, all mental faculties function in such strict coordination and unity that they appear to be different aspects of one distinct entity, called the *mind*. Our innate world seems to revolve around a central aspect of the mind, called the ‘sense of self’ or the ‘innate I’ or the ‘innermost ego,’ as Sir E. S. Eddington calls it. Despite different opinions on the origin and nature of the mind, our common experience will vouch that ‘*something*’ with the above characteristics is an inherent part of human existence. Not knowing the full nature of something does not necessitate and justify the denial of the existence of that thing. The mind is what we all think it is.

*Knowledge* exists in a person’s mind, and thus it is a *mental phenomenon*. We cannot see knowledge since it is not matter but meaning, and therefore a subjective entity, like the mind itself. Currently, no technology exists to retrieve the knowledge (including memory) of a live or dead person since neuroscience cannot tell us the mode and the code of registering. Therefore, there is a need for an intermediary medium for transmitting knowledge as information from one mind to another through a transmission medium, such as spoken language. The act of *conversation* is the process of *conveying meanings* from one mind to another, with sound waves in the air acting as the physical carrier.

In the case of humans, the phrase ‘*retrieve from memory*’ should also be taken with a grain of salt since, unlike an ordinary memory chip, there appears to be no physical storage media in the brain which changes states as experiences are recorded, and no identifiable mechanism of retrieval. This may come as a surprise, but memory appears to be a form of *virtual reality*, like imagination and dreams. That is, all our experiences are recorded and stored in a *virtual cloud* out there. Recent research revealed that remembering the past and imagining the future activate the same brain regions, which suggests that a common brain network underlies both memory and imagination [53,54]. It appears that memory, imagination, and dreams are woven of the same fabric by the same virtual mechanism.

The mind accesses the *memory* by simply willing and directing attention to the intended experience. Yet often memories pop up in the mind on their own, with no intention. The breakdown of the virtual mechanism to access memory and thus the inability to recall past experiences is called *amnesia*. Unlike the memory sticks or hard disks of a computer, we cannot erase our memories. But some memories seem to fade unless they are rejuvenated by intent and attention.

One of the favorite topics of discussion of futurists is the prospect of *downloading the information* that resides in the memory of a terminally ill person and storing it for future use [55]. When technology advances to the point of making artificial brains, the stored memory is to be *uploaded* to a new body. This will supposedly enable the person to live on from where they left off since the *essence of a person* is presumed to be information stored in memory. This overly simplistic notion which stems from the brain-comput5er metaphor misses the point that the brain and conscious mind combination is much more than a *software-loaded microprocessor*, such as a smartphone.

We can download or transmit only the *symbols of information* via physical media, and not the subjective *meaning* which is the essence of information. This means no stored information (more correctly, symbols of information) will acquire meaning unless it is linked to and processed by a conscious mind. Therefore, unless the futuristic technological body comes *equipped with a conscious mind* and closely resembles a live person who suffered amnesia, the notion of downloading and uploading memory may turn out to be just a delusion.

We may lose all or part of our memory as a result of *brain injury*, like losing consciousness, but this does not mean that memory and consciousness are the creations of the brain, which is a lump of fatty meat that weighs about 1.3 kg and whose electrical activity resembles the electrical activity in a microprocessor. We cannot access YouTube videos when our smartphone is damaged, either; but this does not mean that the content of YouTube videos is the creation of our smartphone. The intense electrical activity in the millions of circuits in a smartphone’s microprocessor cannot generate a single frame of a meaningful picture or a trace of consciousness by its own doing. Correlation is not causation. All causal links must be in good functioning order for a process to proceed. The failure of just one of the many links is sufficient to bring the entire process to a halt. The malfunction of a light switch can cause a room to go dark, and repairing it can get the light back. But this does not mean that the light switch is the maker of light.

## 8. Signal Transmission and Information

Misunderstandings associated with information are not limited to just confusing *symbols* of information with the *information* itself. The *signals* of information are often treated as the *information itself*. When we press the A/C button at the control panel of a car, for example, all we do is generate an electric signal routed to the input gates of the car’s microprocessor, which generates a signal to turn the air conditioning device on. Therefore, the microprocessor of a car simply processes *signals* per instructions in its software, not *information*—the microprocessor has no idea of what it is doing. But the conscious and knowledgeable makers and users of the cars refer to this as ‘information processing’ since those signals correspond to information in their minds. The intense electrical signal activity in a microprocessor would not correspond to any information if there were no conscious and knowledgeable beings like humans. This would be evident if all humans were to vanish.

Natural processes—such as radioactive decay and the sun giving off photons—generate tremendous amounts of signals, but no information since no mind is involved. Sunlight striking solar PV cells, for example, generates electric signals due to the photoelectric effect, which can be collected as electrical energy and fed into the electric grid. But no information is transmitted with those signals over the grid. Likewise, neurons in the brain fire electric signals to other neurons through the synapses when their electric charge exceeds a threshold value. But again, this is a purposeless natural signal emission just like radioactive decay, and there is no information associated with it. *Computers* are merely signal processors: They receive signals, process signals, and deliver signals as outputs. Information is in the minds of those who interpret the signals and make sense of them.

When *light* strikes the retina in our eyes, a natural electric signal is generated and is transmitted to the brain cells. It carries no more information than an electric signal generated when sunlight strikes a solar PV cell since there is no meaning ascribed to either. Then it follows that the intense electric activity in the brain is simply a purposeless maze of electrical activity and not information processing. So, it is no surprise that when a person is sleeping, fainted or in a coma, there is no easing down of the brain’s electric activity. But this signal activity cannot be called information processing since there is no consciousness and thus no ascription of meaning involved. Perhaps we should reconsider the current practice of turning a blind eye to the enigmatic mind because we have no idea how to deal with it. An unprejudiced fresh approach stripped off presuppositions is needed to unearth some deep mysteries and get a more factual picture of reality.

The *brain waves* generated can also serve as the medium of transmission of information since certain waveforms correspond to certain meanings. The mere act of intentional thinking of a certain act results in generating the particular electromagnetic waves for that act. Meaning generates its symbols in this case to be received and decoded by the external receivers. In recent years, various *mind-control technologies* have been developed to control devices remotely by brain waves. For example, Toyota created a way of steering a wheelchair by detecting brain waves, without the person having to move a muscle or shout a command [56]. Similarly, toys like remote-controlled helicopters are being developed that fly with mind control by picking up brain signals. Even car control kits are being developed that allow a person to control the moves of a car by thought, with no bodily interference.

## 9. Closing Remarks

Despite the tremendous success of information and communication technologies, it is remarkable that diverse opinions and misconceptions about information abound. The kernel of information is meaning, and the physical symbols of information are just the husk. Without meaning, we cannot speak of information. Yet, under the influence of the ideological conviction of reductionism to reduce everything to matter, the symbols of information are often treated as the information itself in the scientific community at large. This is a deeply entrenched delusion propagated by the observation that meaning always accompanies symbols. We usually take meaning for granted since it is a mental rather than physical phenomenon, and often it is not even recognized as a legitimate form of existence. Somehow meaning is perceived as something generated by the symbols, which is absurd since symbols are merely clusters of senseless letters, sounds, signs, etc.

Perhaps this is not surprising since there are numerous competing theories of mind, and some reductionist materialistic theories view the mind and its attributes such as consciousness and free will as illusions generated by the brain [57]. But this is an ideological view rather than a scientific fact. Such conjectures are the natural extensions of limiting existence to matter, and the dogmatic presumption that all existence must originate from matter and be reduced to matter. When presuppositions are put aside, it is possible to develop a collective view that the ontologically subjective entities of mind, meaning, and consciousness (and even life) are indubitable realities, in line with our shared experiences. Again, not knowing the nature of something is not a valid justification for denying the existence of that thing. After all, we do not know the natures of dark matter and dark energy which constitute 95 percent of the physical realm, either. But we recognize their existence because of the apparent influences they exert.

To summarize, the essence of both knowledge and information is meaning. Information is meaning represented by physical symbols, while knowledge is meaning that resides in a conscious mind. There cannot be knowledge without consciousness since one cannot claim the existence of something unless they are aware of that thing. The symbols of information are the physical media of representation. Signals of information, together with symbols of information, are the means of transmitting information.

Information in the true sense exists only when the symbols of information interact with a conscious mind during an event. We cannot speak of information without a conscious mind since meaning is an ascribed and acquired quality associated with a mind, and not an inherent property of the symbols. We cannot transmit meaning from one mind to another without a common protocol or convention between the symbols and meanings, either.

A concise understanding of information can be established only on the common platform of the mind, meaning, and symbols trio. Moreover, information is not an active agent since it has no causal power and thus no capacity to organize a physical system.

The proposed intuitive mind-centered depiction of information qualifies as a scientific postulation since it is logically consistent, conforms to observations, and explains the commonly observed phenomena well. The depiction of information here as meaning ascribed to symbols is a plausible scientific proposition since it is falsifiable. For example, if it is demonstrated that symbols of information acquire meaning of their own, like different assemblies of matter acquiring different properties such as hardness and conductivity, then this proposition will be deemed falsified. Until this is done, the portrayal of information as depicted here will continue to be a contender among other propositions in information science.

It remains to be seen whether this proposition may serve as a common platform to unify at least some aspects of the existing definitions and descriptions of information. Information and the related concepts cross many disciplines from information science, neuroscience, computer science, and communications to biology and philosophy. Thus, future information studies can be undertaken with a multidisciplinary approach. Several issues touched upon here for completeness, such as the relation of information to consciousness, mind, and life, will benefit immensely from in-depth investigations by multidisciplinary teams.

## Data Availability

Not applicable.
